# The influence of somatostatin analogues on the incidence of pancreatic fistulas and postoperative morbidity in patients undergoing pancreatic resection: A Bayesian network meta-analysis

**DOI:** 10.1371/journal.pone.0331909

**Published:** 2025-09-19

**Authors:** Zonghao Hou, Shengxiang Hou, Zhixin Wang, Haijiu Wang, Manjun Deng, Haining Fan

**Affiliations:** 1 Department of Hepatopancreatobiliary Surgery, Affiliated Hospital of Qinghai University, Qinghai, P. R. China; 2 Qinghai Research Key Laboratory for Echinococcosis, Qinghai, P. R. China; 3 Laboratory of Stem Cell and Regenerative Medicine, Affiliated Hospital of Qinghai University, Qinghai, P. R. China; Kimura Hospital, JAPAN

## Abstract

**Background:**

Pancreatic resection is a critical treatment for pancreatic cancer and other pancreatic diseases. Somatostatin analogs are commonly used to prevent complications following pancreatic resection, but their efficacy and safety remain debated.

**Methods:**

Following PRISMA guidelines, a systematic search was conducted across multiple databases, including PubMed, EMBASE, Scopus, Cochrane Library, Ovid, ClinicalTrials.gov, Web of Science, CNKI, and WanFang Data. The search focused on studies comparing the use of somatostatin analogs after pancreatic surgery. Key outcomes included postoperative pancreatic fistula (POPF), clinically relevant POPF (CR-POPF), mortality, and morbidity. Statistical analysis was performed using a consistency model, calculating relative risk ratios (RR) with 95% confidence intervals (CI), and the Grading of Recommendations Assessment, Development, and Evaluation (GRADE) tool was used to assess the quality of evidence.

**Results:**

In the absence of stratification based on the surgical procedure, For POPF prevention, pasireotide showed a relative risk (RR) of 0.46 (95% CI: 0.23, 0.87, Low) compared to placebo, and octreotide had an RR of 0.76 (95% CI: 0.66, 0.88, Moderate). Somatostatin and vapreotide showed no significant differences. In preventing CR-POPF, pasireotide had an RR of 0.46 (95% CI: 0.23, 0.86, Low), somatostatin had an RR of 0.60 (95% CI: 0.36, 0.99, Moderate), and octreotide had an RR of 0.61 (95% CI: 0.39, 0.94, Moderate). Regarding postoperative mortality, vapreotide showed an RR of 0 (95% CI: 0.00, 0.29, Low), while octreotide, somatostatin and pasireotide did not demonstrate significant effects. For reducing morbidity, octreotide had an RR of 0.74 (95% CI: 0.66, 0.82, Moderate), somatostatin had an RR of 0.76 (95% CI: 0.66, 0.87, Moderate), vapreotide and pasireotide showed no significant effect.In Pancreaticoduodenectomy subgroup, somatostatin showed an RR of 0.22(95% CI: 0.03, 0.84, Moderate) for preventing CR-POPF.For all the other outcomes, neither somatostatin nor octreotide proved effective.

**Conclusion:**

While robust evidence confirms the efficacy of octreotide in preventing POPF, a critical concern regarding its inconsistent efficacy within the PD subgroup persists. This variability indicates that the overall clinical benefit of octreotide may be predominantly attributable to its utility in non-PD pancreatic resections.

## Introduction

Pancreatic resection is the standard surgical procedure for treating pancreatic cancer and other pancreatic diseases. However, it is a complex operation that is often accompanied by severe postoperative complications and long-term challenges. Postoperative pancreatic fistula (POPF) is one of the most common and severe complications, with an incidence ranging from 13% to 41%. POPF occurs when pancreatic fluid leaks, leading to potentially severe outcomes such as sepsis and multiple organ failure [1,2]. It can also significantly impact patient prognosis by delaying adjuvant chemotherapy or reducing overall survival [3].

In recent years, somatostatin analogs have gained attention for their potential to prevent postoperative complications, especially POPF, following pancreatic resection. Previous studies have explored the effectiveness of somatostatin analogs in preventing POPF. For example, a phase II clinical trial demonstrated that somatostatin reduced the incidence of clinically relevant postoperative pancreatic fistulas compared to historical controls [4,5]. However, another study found that combining somatostatin analogues with corticosteroids did not significantly reduce the incidence of clinically relevant POPF (CR-POPF) in high-risk patients [6]. Additionally, a systematic review and meta-analysis concluded that while somatostatin analogs helped reduce postoperative morbidity and the incidence of POPF, they had no significant effect on mortality or hospital stay duration [7]. Despite these findings, the clinical application of somatostatin analogs remains controversial, suggesting that their efficacy in preventing complications after pancreatic resection may vary.

Given the variety of somatostatin analogs available for clinical use, robust evidence is needed to guide treatment decisions. A network meta-analysis of existing data can compare the efficacy of different interventions, synthesizing a broader range of evidence to provide deeper insights into their relative advantages and disadvantages. Therefore, this study aims to systematically review and conduct a network meta-analysis to compare the efficacy of various somatostatin analogs in preventing short-term complications following pancreatic resection, offering valuable information for clinical practice.

## Methods

### Study design

This study follows the Preferred Reporting Items for Systematic Reviews and Meta-Analyses (PRISMA) guidelines and the PRISMA-2020 Statement, as well as the PRISMA-Network Meta-Analysis (NMA) extension statement [8]. The study protocol was registered at PROSPERO (Registration No. CRD42025634241).

### Literature search

A three-step search was conducted by three independent researchers to identify all potentially eligible studies published from database inception to November 23, 2024. First, a systematic search was performed across PubMed, Embase, Ovid, Web of Science, and the Cochrane Central Register of Controlled Trials. Predefined search terms included “randomized controlled trial,” “pancreas,” “pancreatic cancer,” “pancreatic surgery,” “somatostatin,” “somatostatin analogues,” “octreotide,” “lanreotide,” “pasireotide,” and “vapreotide” (specific search strategies are detailed in S2 Table in S1 File). Second, to maximize data availability, we also searched various clinical trial registration websites and professional journals (S3 Table in S1 File) for ongoing, unpublished, and potential trials. Relevant systematic reviews and guideline references were also considered. Reference management was done using EndNote 21 software, and duplicates were removed. There were no language restrictions, and studies for which full texts could not be obtained were excluded. The search was independently conducted by three researchers, and any disagreements were resolved through consultation with a fourth reviewer.

### Data extraction and quality assessment

This study adhered to the PRISMA 2020 flowchart for the literature selection process. Three independent reviewers evaluated the titles, abstracts, and full texts to determine whether the studies met the inclusion and exclusion criteria based on the PICO (Population, Intervention, Comparison, Outcome) principles (S4 Table in S1 File). A fourth reviewer consolidated the results, and a fifth reviewer resolved any disagreements. Data extraction followed a standardized form based on the Cochrane Handbook for Systematic Reviews of Interventions [9]. This form collected details about the study’s authors, publication year, patient region, interventions, measurement methods, intervention duration, and all recorded clinical outcomes. For the purpose of facilitating image analysis and subsequent result interpretation, the placebo and blank control groups were consolidated. Two independent researchers entered the relevant data into electronic spreadsheets, and a third researcher conducted a final check to ensure accuracy. An expert group, consisting of five liver and biliary surgeons at the associate professor level or higher, was assembled to determine the key outcomes. The expert group agreed that postoperative pancreatic fistula (POPF) (according to the ISGPF definition) and clinically relevant POPF (CR-POPF) should be the primary outcomes, while mortality and morbidity rates were considered secondary outcomes.

### Bias analysis and quality assessment

The quality of the included studies was assessed using the Cochrane Risk of Bias Tool (version 2.0) [10], based on five domains: risk of bias in the randomization process, bias from blinding implementation of randomized interventions [11], bias due to missing outcome data, bias in outcome measurement, and bias in the selection of reported results. Studies were classified as low risk if all five domains were rated as low risk, as high risk if at least one domain was rated as high risk, and as unclear risk for the remaining studies. The Grading of Recommendations Assessment, Development, and Evaluation (GRADE) tool was used to assess the quality of evidence based on five factors: limitations, indirectness, inconsistency, imprecision, and publication bias. Evidence quality was rated from high to low: high, moderate, low, and very low. Initial GRADE ratings were high, with adjustments made according to the aforementioned factors. Inconsistency was assessed in terms of both heterogeneity and consistency, with potential downgrades of up to two levels, while other factors could only be downgraded by one level. Detailed information is provided in S5 Table in S1 File. All study outcomes were independently assessed by two reviewers, with discrepancies resolved through consensus.

### Statistical analysis

A network diagram was created using Stata 17.0 software to visually represent the relationships between different interventions. The size of the circles indicates the sample size of each intervention, while the width of the lines indicates the number of studies comparing those interventions. In some studies, the control group did not use a placebo, but for consistency in analysis and presentation, these control groups were treated as if they had used a placebo. Direct and indirect comparisons were conducted in a Bayesian framework using the Markov Chain Monte Carlo method. Statistical analysis was performed with R software (version 4.3.1) and JAGS software (version 4.3.1) for modeling. The “gemtc,” “rjags,” “openxlsx,” and “forestploter” R packages were used for statistical analysis and data output. The model settings included 6 chains, an initial value of 2.5, 50,000 adaptation iterations, 200,000 simulation iterations, and a thinning factor of 10 to calculate estimates of combined sensitivity and specificity with 95% credible intervals (CIs). The final results were presented as risk ratios (RR). The convergence of the model was assessed using the R-hat value, where an R-hat value greater than 1.05 may indicate potential convergence issues. Bayesian network meta-analysis provided overall ranking probabilities for each intervention, allowing rankings from best to worst. Rankings were visualized using the surface under the cumulative ranking curve (SUCRA), where higher SUCRA values indicate a higher likelihood of being ranked better. Since heterogeneity is inevitably present between studies, node-splitting methods were used to assess local consistency by comparing direct and indirect comparisons, with p-values used to assess differences. If p-values in direct and indirect comparisons were greater than 0.05, heterogeneity was considered non-significant. A consistency model was then used for subsequent statistical analyses. Pairwise and network heterogeneity were evaluated using I²; values above 50% indicated significant heterogeneity. Finally, forest plots were used for visualizing the results [12]. Funnel plots were also employed to assess publication bias [13].

## Results

### Search results

**Fig 1:**
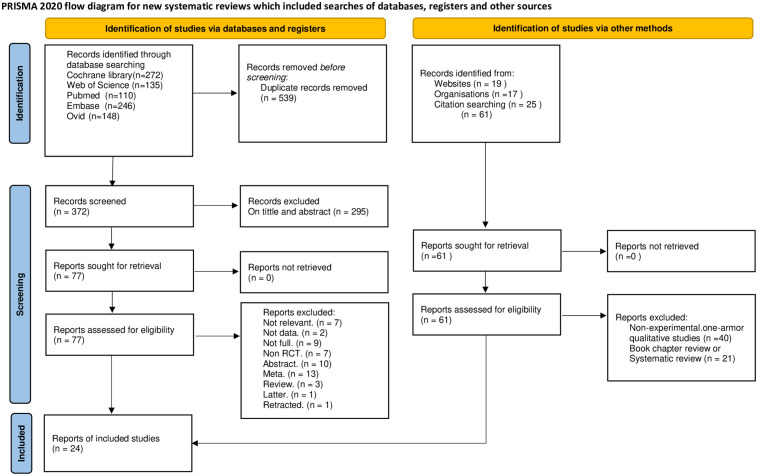
Literature search and selection flowchart.

A total of 911 articles were identified through the search strategy. Of these, 539 were duplicates. After screening titles, keywords, and abstracts, 295 studies were excluded, leaving 77 articles for full-text review. Following the full-text review of these 77 articles, 53 were excluded for various reasons, as detailed in S6 Table in S1 File. Ultimately, 24 studies involving 4050 participants were included in the analysis. The PRISMA flowchart of the literature search is shown in Fig 1. These studies included 16 trials on octreotide [14–29], 7 trials on somatostatin [29–35], 1 trial on pasireotide [36], and 1 trial on vapreotide [37]. Within this cohort, the distribution of interventions across the included studies was as follows: 12 trials involved the Pancreaticoduodenectomy (PD) group, 10 trials examined the PD in conjunction with distal pancreatectomy (DP), 1 trial assessed Pancreas Transplantation, and 1 trial incorporated a group receiving both pylorus-preserving pancreaticoduodenectomy (PPPD) alongside PD and DP procedures. The main characteristics of the included studies are summarized in Table 1. In terms of bias risk, 15 trials were classified as unclear risk and 9 trials as high risk according to the Cochrane Risk of Bias Tool, as detailed in S1 Fig in S1 File.

**Table 1:** Characteristics of the included studies.

Study	Year	Drug.a	Drug.b	Somatostatin-analogue	Definition of POPF	Blinding	Surgery	Outcome	Risk of bias
Büchler [14]	1992	octreotide	placebo	Perioperative octreotide 100 μg 3 × day for 7 days	Amylase and lipase >3 times serum concentration, > 3 days postop, > 10 ml/h	Double	PD + DP	①③④	Unclear
Pederzoli [15]	1994	octreotide	placebo	Perioperative octreotide 100 μg 3 × day for 7 days	>10 ml/day for >4 days after POD 4, amylase >3 times normal	Double	PD + DP	①③④	Unclear
Friess [16]	1995	octreotide	placebo	Perioperative octreotide 100 μg 3 × day for 7 days	Amylase and lipase >3 times serum level, > 3 days postop, > 10 ml/h	Double	PD + DP	①③④	Unclear
Montorsi [17]	1995	octreotide	placebo	Perioperative octreotide 100 μg 3 × day for 7 days	>10 ml/day fluids exceeding 3 times normal serum amylase after POD 3	Double	PD + DP	①③④	Unclear
Lowy [18]	1997	octreotide	placebo	Perioperative octreotide 100 μg 3 × day for 5 days	drainageof amylase-rich fluid (>2.5 times the upper limit of normal for serum amylase) and clinical signs or the need of reintervention	Na	PD	①②③④	High
Benedetti [19]	1998	octreotide	placebo	Perioperative octreotide 100 μg 3 × day for 5 days	Na	Na	PT	③	Unclear
Yeo [20]	2000	octreotide	placebo	Perioperative octreotide 250 μg 3 × day for 7 days	>50 ml/day fluids exceeding >3 times normal serum value on or after day 10 or radiological sings of POPF	Double	PD	①③④	Unclear
Gouillat [30]	2001	somatosta	placebo	Perioperative somatostatin 250 μg/h for 7 days	>100 ml/day exceeding 5 times normal serum amylase after POD 3, persisting after POD 12 or in association with clinically relevant symptoms requiring surgery, drainage or intensive care	Double	PD	①②③④	Unclear
Shan [34]	2003	somatosta	placebo	Perioperative somatostatin 250 μg/h for 7 days	an elevation of serum amylase from POD 4, with morphologic evidence by CT, or laparotomy	Single	PD	①③④	High
Sarr [37]	2003	vapreotide	placebo	Perioperative vapreotide 600 μg 2 × day for 7 days	>30 ml/day 5, amylase or lipase >5 times normal	Double	PD + DP	①③④	Unclear
Suc [21]	2004	octreotide	NA	Perioperative octreotide 100 μg 3 × day for 10 days	Fluids exceeding >4 times normal serum level or any radiological signs of POFP	Single	PD + DP	①③④	High
Hesse [22]	2005	octreotide	NA	Perioperative octreotide 100 μg 3 × day for 7 days	>100 ml/day exceeding 5 times normal serum amylase after POD 3, persisting after POD 12 or in association with clinically relevant symptoms requiring surgery, drainage or intensive care	Open	PD + DP	①③④	High
Shan [33]	2005	somatostati	NA	Perioperative somatostatin 250 μg/h for 7 days	>10 ml/day fluids exceeding amylase >3 times serum level for >7 days	Na	PD	①③	Unclear
Kollmar [23]	2008	octreotide	placebo	Perioperative octreotide 100 μg 3 × day for 7 days	ISGPF	Double	PD	①③	Unclear
Katsourakis [31]	2010	somatostatin	NA	Perioperative somatostatin 250 μg/h for 7 days	amylase> 3 × plasma amylase after POD 3,	Open	PPPD+ PD + DP	①④	High
Fernández-Cruz [24]	2013	octreotide	placebo	Perioperative octreotide 100 μg 3 × day for 10 days	ISGPF	Na	PD	②③④	High
Katsourakis [35]	2013	somatostatin	NA	Perioperative somatostatin 250 μg/h for 6 days	ISGPF	Open	PD + DP	①③④	Unclear
Allen [36]	2014	pasireotide	placebo	Perioperative pasireotide 900 μg 2 × day for 7 days	ISGPF	Double	PD + DP	①②③④	Unclear
Kurumbo [25]	2015	octreotide	NA	Perioperative octreotide 100 μg 3 × day for 6 days	ISGPF	Open	PD	①②③④	High
Kong [26]	2016	octreotide	placebo	Perioperative octreotide 100 μg 3 × day for 10 days	amylase> 3 × plasma amylase after POD 3,	NA	PD	①②③④	Unclear
El Nakeeb [27]	2018	octreotide	placebo	Perioperative octreotide 100 μg 3 × day untl time of resumption of oral fluids intake	ISGPF	Na	PD	①②③④	Unclear
You [28]	2019	octreotide	NA	Perioperative octreotide 100 μg 3 × day untl time of resumption of oral fluids intake	Na	Single	PD	①②③	High
Cao [32]	2021	somatostatin	NA	Perioperative somatostatin 250 μg/h > 120h	ISGPS	Na	PD	①③④	Unclear
Gaujoux [29]	2024	Somatostatin	Octreotide	Perioperative octreotide 100 μg 3 × day for 10 days,somatostatin 250 μg/h for 6 days	ISGPF	Open	PD + DP	①②③④	High

PD: Pancreaticoduodenectomy; DP: Distal Pancreatectomy; PT:Pancreas Transplantation;PPPD:Pancreaticoduodenectomy.Outcome:①: postoperative pancreatic fistula:②: Clinical Relevant postoperative pancreatic fistula③: Mortality:④: Morbidity.

### Network diagram

**Fig 2:**
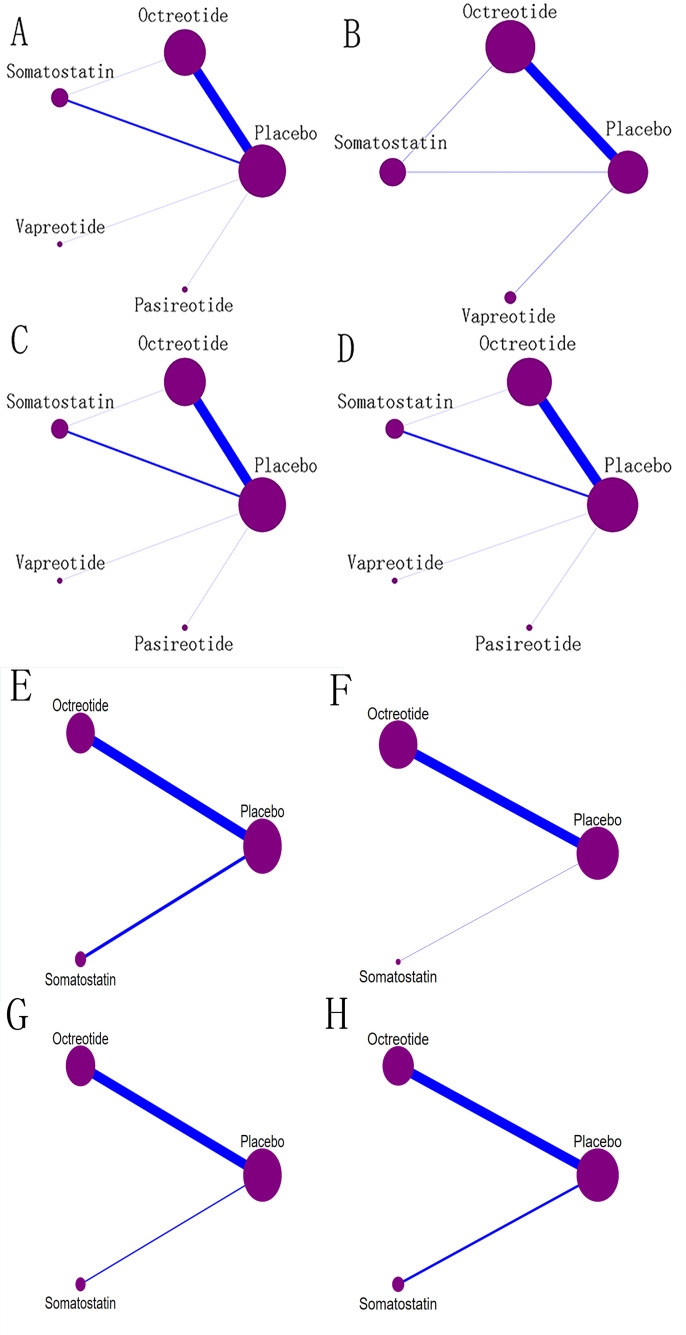
Network plot of outcome indicators. (A) POPF of all studies (B) CR-POPF of all studies (C) Mortality of all studies (D) Morbidity of all studies (E) POPF of PD subgroup (F) CR-POPF of PD subgroup (G) Mortality of PD subgroup (H) Morbidity of PD subgroup.

This study focused on four clinical outcomes. The network diagram (Fig 2) visually represents the relationships between different interventions. In the diagram, the width of the lines is proportional to the number of trials involved in each pairwise treatment comparison, while the size of the circles corresponds to the number of participants (sample size) randomly assigned to each intervention.

### Network meta-analysis

#### Postoperative Pancreatic Fistula (POPF).

**Fig 3:**
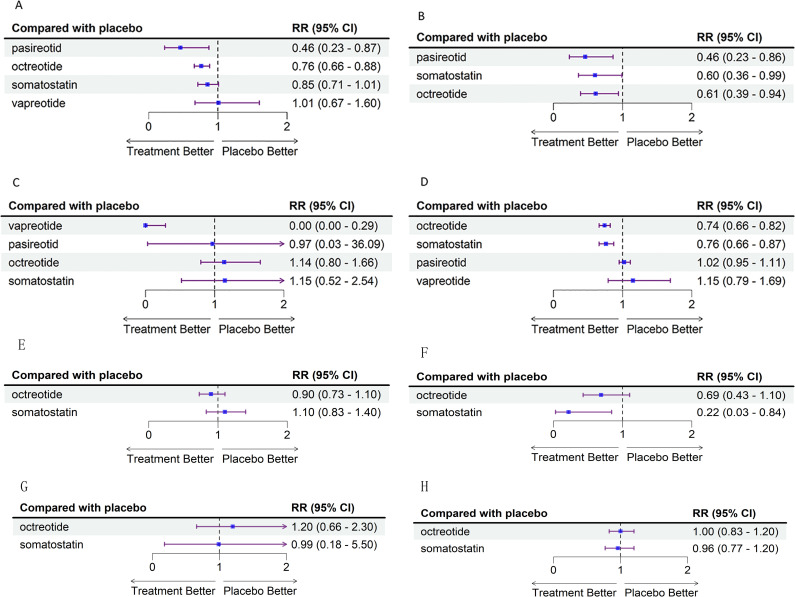
Forest plot of the network meta-analysis. (A) Forest plot of the network meta-analysis for (A) POPF of all studies (B) CR-POPF of all studies (C) Mortality of all studies (D) Morbidity of all studies (E) POPF of PD subgroup (F) CR-POPF of PD subgroup (G) Mortality of PD subgroup (H) Morbidity of PD subgroup in all trials. Somatostatin analogs are compared with placebo, which serves as the reference compound. RR = Risk Ratio. CI = Confidence Interval.

**Fig 4:**
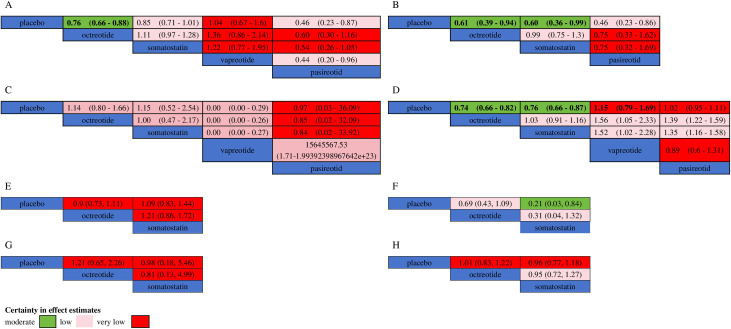
Head-to-head comparison of therapy. (B) Head-to-head comparison of (A) POPF of all studies (B) CR-POPF of all studies (C) Mortality of all studies (D) Morbidity of all studies (E) POPF of PD subgroup (F) CR-POPF of PD subgroup (G) Mortality of PD subgroup (H) Morbidity of PD subgroup for various drugs. Data are presented as RR (95% CI) for column-defined treatment compared with row-defined treatment. The quality of evidence (according to GRADE) is incorporated into the figure. RR = Risk Ratio. CI = Confidence Interval. Effect sizes with statistically significant differences are bolded, and the color of each cell represents the certainty of the effect estimate.

**Fig 5:**
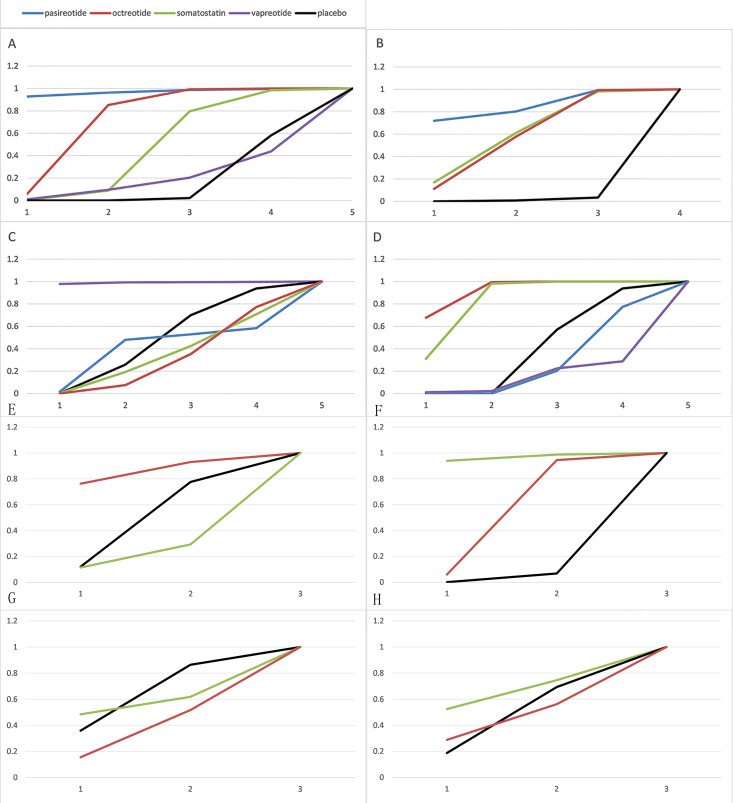
SUCRA ranking of intervention effectiveness Annotation. SUCRA rankings for (A) POPF of all studies (B) CR-POPF of all studies (C) Mortality of all studies (D) Morbidity of all studies (E) POPF of PD subgroup (F) CR-POPF of PD subgroup (G) Mortality of PD subgroup (H) Morbidity of PD subgroup.

POPF was reported in 22 studies involving 3,975 patients, with five different interventions included. As shown in Figs 3A, 4A, and 5A, analysis using a fixed-effect model revealed that pasireotide demonstrated a relative risk (RR) of 0.46 (95% CI: 0.23, 0.87) compared to placebo, with a SUCRA value of 0.97. Octreotide showed an RR of 0.76 (95% CI: 0.66, 0.88), with a SUCRA value of 0.73. Within the PD subgroup, octreotide showed an RR of 0.90 (95% CI: 0.73, 1.10), with a SUCRA value of 0.85. Somatostatin had an RR of 1.10 (95% CI: 0.83, 1.40), with a SUCRA value of 0.20. Somatostatin had an RR of 0.85 (95% CI: 0.71, 1.01), with a SUCRA value of 0.47, and vapreotide had an RR of 1.01 (95% CI: 0.67, 1.60), with a SUCRA value of 0.19.

The overall heterogeneity was low (I² = 34%), indicating good consistency among the included studies, which supports the reliability of the network meta-analysis results. Funnel plots did not show significant publication bias (S2 Fig A in S1 File).

#### Clinically Relevant Postoperative Pancreatic Fistula (CR-POPF).

CR-POPF was reported in 9 studies involving 1,774 patients, with four interventions included. As shown in Figs 3B, 4B, and 5B, analysis using a fixed-effect model revealed that pasireotide showed an RR of 0.46 (95% CI: 0.23, 0.86), with a SUCRA value of 0.84. Somatostatin had an RR of 0.60 (95% CI: 0.36, 0.99), with a SUCRA value of 0.59, and octreotide showed an RR of 0.61 (95% CI: 0.39, 0.94), with a SUCRA value of 0.56. Within the PD subgroup, octreotide showed an RR of 0.69(95% CI: 0.43, 1.10), with a SUCRA value of 0.50. Somatostatin had an RR of 0.22 (95% CI: 0.03, 0.84), with a SUCRA value of 0.96. The overall heterogeneity was low (I² = 2%), indicating high consistency among the included studies, supporting the reliability of the network meta-analysis results. Funnel plots did not indicate significant publication bias (S2 Fig B in S1 File).

#### Mortality.

Mortality was reported in 23 studies involving 3,987 patients and included five interventions. As shown in Figs 3C, 4C, and 5C, analysis using a fixed-effect model revealed that vapreotide had an RR of 0 (95% CI: 0.00, 0.29), with a SUCRA value of 0.99. Pasireotide had an RR of 0.97 (95% CI: 0.03, 36.09), with a SUCRA value of 0.40. Somatostatin showed an RR of 1.15 (95% CI: 0.52, 2.54), with a SUCRA value of 0.33, and octreotide had an RR of 1.14 (95% CI: 0.80, 1.66), with a SUCRA value of 0.30. Within the PD subgroup, octreotide showed an RR of 1.20(95% CI: 0.66, 2.30), with a SUCRA value of 0.0.34. Somatostatin had an RR of 0.99 (95% CI: 0.18, 5.50), with a SUCRA value of 0.55. The overall heterogeneity was low (I² = 0%), indicating high consistency among the studies, supporting the reliability of the network meta-analysis results. Funnel plots did not show significant publication bias (S2 Fig C in S1 File).

#### Morbidity.

Morbidity was reported in 20 studies involving 3,888 patients and included 20 interventions. As shown in Figs 3D, 4D, and 5D, analysis using a fixed-effect model revealed that octreotide had an RR of 0.74 (95% CI: 0.66, 0.82), with a SUCRA value of 0.92. Somatostatin had an RR of 0.76 (95% CI: 0.66, 0.87), with a SUCRA value of 0.82. Pasireotide had an RR of 1.02 (95% CI: 0.95, 1.11), with a SUCRA value of 0.24, and vapreotide had an RR of 1.15 (95% CI: 0.79, 1.69), with a SUCRA value of 0.14. Within the PD subgroup, octreotide showed an RR of 1.00(95% CI: 0.83, 1.20), with a SUCRA value of 0.43. Somatostatin had an RR of 0.96 (95% CI: 0.77, 1.20), with a SUCRA value of 0.63. The overall heterogeneity was low (I² = 0%), indicating high consistency among the studies, supporting the reliability of the network meta-analysis results. Funnel plots did not indicate significant publication bias (S2 Fig D in S1 File).

#### Quality of evidence....

We used the GRADE methodology to assess the quality of the effectiveness estimates for each comparison. Effectiveness and certainty estimates are presented in league matrices, with detailed processes outlined in S7 Table in S1 File. As shown in Fig 4, octreotide demonstrated the best statistical effects in reducing POPF, CR-POPF, and morbidity, with evidence quality assessed as moderate confidence. Somatostatin showed better statistical effects in reducing CR-POPF and overall morbidity, with evidence quality rated as low and moderate confidence, respectively. Vapreotide showed the best statistical effect in reducing mortality, but the evidence quality was assessed as low. Pasireotide achieved the highest statistical effect values and SUCRA scores for reducing POPF and CR-POPF, but the evidence quality was rated as low.

Within the PD subgroup, the evidence quality was rated as moderate for somatostatin and low for octreotide concerning the prevention of CR-POPF. For all other outcomes assessed, the quality of evidence for both somatostatin and octreotide was rated as very low.Finally, forest plots visually presented the results of the node-splitting method used to assess consistency testing (S3 Fig in S1 File). No significant inconsistencies were observed after evaluating direct comparisons, indirect comparisons, and network comparisons.

#### Sensitivity analysis.

In the course of our sensitivity analysis, distinct network analyses were executed for the blank control cohort and the placebo cohort. A subsequent comparison with the pooled cohort revealed no statistically significant discrepancies between the findings of the separate analyses and those of the combined group.(S9 Table in S1 File).

## Discussion

This study is based on 24 randomized controlled trials (RCTs) involving 4,054 patients who were randomly assigned to one of four somatostatin analog groups or a placebo group. It encompasses four somatostatin analogs and provides data for head-to-head comparisons. Compared to previous meta-analyses, this analysis is more comprehensive as it includes not only four active treatment regimens and a placebo but also conducts network comparisons and employs the GRADE method to assess the quality of the conclusions. These findings provide strong support for the development of future related guidelines.

In this meta-analysis, we observed significant differences in the efficacy of several somatostatin analogs in preventing postoperative pancreatic fistula (POPF) compared to placebo. Only pasireotide and octreotide demonstrated superior efficacy. Although pasireotide was rated as having low confidence by GRADE, it showed better results than previously reported in a meta-analysis that suggested it was ineffective in reducing POPF incidence [38]. Additionally, somatostatin and vapreotide did not show a significant difference in preventing POPF. For the prevention of clinically relevant POPF (CR-POPF), all somatostatin analogs demonstrated promising efficacy, with only octreotide rated as having moderate confidence by GRADE. In reducing mortality, only vapreotide showed superior efficacy with a significant difference. However, due to the inclusion of only one study, there is a risk of bias, leading to a lower GRADE rating. In reducing morbidity, octreotide and somatostatin demonstrated better efficacy, both rated as having moderate confidence by GRADE. The efficacy of somatostatin in reducing postoperative complications is consistent with the findings of Schorn et al.‘s meta-analysis [39]. Notably, despite being rated as having very low confidence by GRADE, vapreotide increased the risk of postoperative complications following pancreatectomy. Within the subgroup analysis concerning Pancreaticoduodenectomy (PD), both somatostatin and octreotide were subjected to evaluation. The findings indicated that only somatostatin exhibited efficacy specifically in the prevention of postoperative pancreatic fistula with clinical relevance (CR-POPF), evidenced by a Risk Ratio (RR) of 0.22 (95% Confidence Interval: 0.03–0.84). The GRADE assessment assigned a moderate quality rating to this evidence. Conversely, neither somatostatin nor octreotide demonstrated statistically significant efficacy for the prevention of overall Postoperative Pancreatic Fistula (POPF), the reduction of Mortality, or the mitigation of Morbidity.

The variations in efficacy among different somatostatin analogs may be attributed to their distinct pharmacological mechanisms. Somatostatin analogs act on five types of somatostatin receptors (SSTR1−5) [40,41], with varying affinities leading to differences in side effects and efficacy across drugs. By binding to these receptors, somatostatin analogs inhibit adenylate cyclase activity, reducing cyclic AMP levels, and suppressing the secretion of growth hormone, thyroid-stimulating hormone, and various gastrointestinal hormones [40,42].Octreotide primarily targets SSTR2 and SSTR5 receptors [43], particularly effective in inhibiting growth hormone (GH), thyroid-stimulating hormone (TSH), and gastrointestinal peptides, while also reducing visceral blood flow and gastrointestinal motility [44]. Octreotide’s long half-life (72−110 minutes) allows for stable, sustained effects, making it suitable for long-term treatment [45]. It has been widely used in clinical practice, supported by our research for its efficacy in reducing POPF and postoperative complications.Somatostatin acts on all five somatostatin receptors (SSTR1−5) [40,41], with a higher affinity for SSTR2 and some affinity for SSTR3, but lower affinities for SSTR1 and SSTR4 [40]. Its short half-life necessitates frequent dosing, limiting its clinical application in certain cases. Despite this, it shows some efficacy in reducing postoperative complications.Vapreotide has high affinities for SSTR2 and SSTR5 and blocks the neurokinin-1 receptor (NK1R), which exerts analgesic effects [46]. Despite promising results in reducing mortality in clinical trials, vapreotide’s overall efficacy is weak and it is not effective in reducing POPF or postoperative complications [37], making it unsuitable as a first-line treatment.Pasireotide, a second-generation somatostatin receptor ligand (SRL), primarily targets SSTR1, SSTR2, SSTR3, and SSTR5, with strong affinity for SSTR5 [47,48]. This makes it potentially more potent in inhibiting pancreatic secretion than first-generation SRLs. However, its use is associated with higher rates of hyperglycemia and liver function impairment, necessitating cautious application in high-risk patients, such as those with diabetes or impaired liver function [47,49].Somatostatin analogs, including octreotide, lanreotide, and pasireotide, exhibit a higher cost profile compared to native somatostatin, necessitating meticulous consideration of cost implications in their therapeutic application. Octreotide, for example, is extensively utilized as a somatostatin analog owing to its prolonged duration of action and demonstrated efficacy in the management of conditions such as acromegaly and neuroendocrine tumors. However, this therapeutic benefit is accompanied by a substantial financial burden. In the context of metastatic gastrointestinal neuroendocrine tumors (GI-NET), octreotide long-acting release (LAR) demonstrates a lower cost profile relative to lanreotide. Comparative analyses indicate payment costs for octreotide LAR at $74,566,$180,082, and $262,344, respectively, whereas lanreotide incurred higher expenditures [50]. Conversely, in Colombia, octreotide was ascertained to be more cost-effective for acromegaly treatment than lanreotide, correlating with reduced total healthcare expenditures and superior disease control efficacy [51]. Nevertheless, in Brazil, the elevated acquisition cost of octreotide LAR vials substantially exacerbated the economic strain on the public health system, implying that increased utilization of lanreotide might facilitate cost reduction [52]. Despite their considerable expense, somatostatin analogs remain indispensable in the management of diverse endocrine and non-endocrine disorders, wherein their therapeutic advantages frequently surpass their financial implications [53,54]. Consequently, the cost-effectiveness of these therapeutic agents represents a pivotal factor, particularly within resource-constrained settings where high acquisition costs may impede their accessibility [51]. Thus, a judicious selection of pharmacotherapies that optimally align with both patients’ physiological requirements and economic circumstances is imperative.

The incidence of complications after pancreatectomy is also influenced by various intraoperative management techniques. For instance, a 2022 meta-analysis evaluated the effectiveness of pancreatic duct stent placement in preventing postoperative pancreatic fistulas after pancreaticoduodenectomy. This study found no significant difference in POPF incidence between the stent use group and the non-use group, although further analysis suggested that external stents significantly reduced POPF incidence [55]. Advancements in surgical techniques, such as pancreatic anastomosis and drainage tube placement, have contributed to changes in POPF incidence [56]. These techniques can affect the efficacy of somatostatin analogs, making the combined effect of different treatment modalities important to consider. Despite these advancements, octreotide has shown the most promising efficacy in preventing postoperative complications, and clinicians may consider incorporating it as the preferred somatostatin analog in relevant treatment guidelines.

Our literature search was comprehensive, being the first to compare various somatostatin analogs (including octreotide, somatostatin, pasireotide, and vapreotide), providing clinicians with more robust evidence to guide medication choices. Additionally, we employed Bayesian network meta-analysis methods, which allow for more accurate estimation of the relative efficacy between different interventions and address heterogeneity between direct and indirect comparisons. However, some limitations should be considered when interpreting our results. Certain studies included in our analysis have a risk of bias, which could affect the reliability of the findings. For example, we conducted a stratified analysis stratified by the surgical procedure. However, constrained by the available data, our analysis was restricted to the PD subgroup only. We observed a lack of efficacy within the PD subgroup, whereas efficacy was noted in the unclassified dataset. Furthermore, the scarcity of studies related to Pasireotide and Vapreotide in our analysis may have resulted in an inadequate assessment. Subsequently, it is imperative to conduct additional randomized controlled trials (RCTs) focusing on Pasireotide and Vapreotide to comprehensively evaluate their effectiveness in preventing complications following pancreatic resection. We analyzed only average treatment effects and did not delve into individual patient responses or their clinical and demographic influencing factors, such as age, gender, severity of symptoms, or disease duration. Furthermore, due to a lack of relevant data in the original studies, we were unable to quantify long-term outcomes for postoperative patients.During the course of our investigation, it was observed that there is a paucity of research dedicated to assessing the cost-effectiveness of patient hospitalization and postoperative recovery. This is a significant consideration in areas where resources are limited and medical development is not yet advanced, indicating a necessity for further research to address this deficiency. Despite its constraints, the current meta-analysis provides substantial reference value regarding the utilization of somatostatin analogues post-pancreatectomy. Subsequent research is required to delineate the role of somatostatin analogues in the prevention of complications after pancreatectomy and to formulate more comprehensive guidelines for their use.

## Conclusion

This study utilized Bayesian network meta-analysis to compare the efficacy of various somatostatin analogs in preventing short-term complications following pancreatectomy. The results indicate that octreotide is the most effective in preventing postoperative pancreatic fistula (POPF), clinically relevant POPF (CR-POPF), and morbidity, with high-quality evidence supporting its use. We hypothesize that this discrepancy may be attributable to the effectiveness of somatostatin analogs demonstrated in other surgical modalities. Although this efficacy could be confounded by the bias stemming from the lack of efficacy observed in the PD subgroup, it appears sufficient to counteract this bias. Nevertheless, this remains speculative, and further randomized controlled trials (RCTs) investigating other pancreatic resection techniques are warranted to validate this hypothesis.

## Supporting information

S1 File **S1 Fig.** Quality assessment of the included studies and risk of bias summary. **S2 Fig.** Funnel Char Of Publication Bias. A:POPF;B:CR-POPF;C:Mortality;D:Morbidity. **S3 Fig.** Forest plot for inconsistency testing.A:POPF;B:CR-POPF;C:ortality;D:Morbidity. **S1 Table.** PRISMA 2020 checklist. **S2 Table.**Index and keyword terms used in the databases. **S3 Table.**Lists of clinical trial registries and specialized journals. **S4 Table.**Eligibility criteria. **S5 Table**.Specific meaning of certainty in effect estimates. **S6 Table.**List of excluded studies. **S7 Table**.GRADE Quality Assessment Table for Network Analysis Results. **S8 Table** The dataset utilized for the purposes of this investigation. **S9 Table** Sensitivity Analysis.
(ZIP)
