# Supplementary material for: The influence of somatostatin analogues on the incidence of pancreatic fistulas and postoperative morbidity in patients undergoing pancreatic resection: A Bayesian network meta-analysis
Source: PLoS One. 2025 Sep 19;20(9):e0331909. doi: 10.1371/journal.pone.0331909 (PMC12449010; doi:10.1371/journal.pone.0331909)
Supplement: S1 File — S1 Fig. Quality assessment of the included studies and risk of bias summary. S2 Fig. Funnel Char Of Publication Bias. A:POPF;B:CR-POPF;C:Mortality;D:Morbidity. S3 Fig. Forest plot for inconsistency testing.A:POPF;B:CR-POPF;C:ortality;D:Morbidity. S1 Table. PRISMA 2020 checklist. S2 Table.Index and keyword terms used in the databases. S3 Table.Lists of clinical trial registries and specialized journals. S4 Table.Eligibility criteria. S5 Table.Specific meaning of certainty in effect estimates. S6 Table.List of excluded studies. S7 Table.GRADE Quality Assessment Table for Network Analysis Results. S8 Table The dataset utilized for the purposes of this investigation. S9 Table Sensitivity Analysis. (ZIP) [file pone.0331909.s001.zip › S2_Table.docx]

# **S2 Table.** Index and keyword terms used in the databases.

| Database | Index and keyword terms |
| --- | --- |
| PubMed | 1 (Pancreatectomy[MeSH Terms]) OR (pancreas[Title/Abstract] OR pancreatic cancer[Title/Abstract] OR pancreatic surgery[Title/Abstract] OR Pancreatectomy[Title/Abstract] OR distal pancreatectomy[Title/Abstract] OR Whipple[Title/Abstract] OR Pancreaticoduodenectomy[Title/Abstract] OR pancreatic resection[Title/Abstract] OR Total pancreatectomy[Title/Abstract] OR Partial pancreatectomy[Title/Abstract] OR Pylorus-preserving pancreaticoduodenectomy[Title/Abstract] OR PPPD[Title/Abstract])  2 (Somatostatin[MeSH Terms]) OR (somatostatin[Title/Abstract] OR somatostatin analogues[Title/Abstract] OR octreotide[Title/Abstract] OR lanreotide[Title/Abstract] OR pasireotide[Title/Abstract] OR vapreotide[Title/Abstract])  3 (randomized controlled trial[MeSH Terms]) OR (randomized controlled trial[Title/Abstract] OR RCT[Title/Abstract] OR randomized[Title/Abstract] OR randomised[Title/Abstract] OR random[Title/Abstract])  4 ("Review" [Publication Type] OR "Systematic Review" [Publication Type] OR "Meta-Analysis" [Publication Type] OR "Editorial" [Publication Type] OR "Case Reports" [Publication Type] OR "Comment" [Publication Type])  5 #1 AND #2 AND #3  6 #5 NOT #4 |
| Cochrane | ID Search  #1 MeSH descriptor: [Pancreatectomy] explode all trees  #2 (" pancreas" OR "pancreatic cancer" OR "pancreatic surgery" OR "Pancreatectomy" OR "distal pancreatectomy" OR "Whipple" OR "Pancreaticoduodenectomy" OR "pancreatic resection" OR "Total pancreatectomy" OR "Partial pancreatectomy" OR "Pylorus-preserving pancreaticoduodenectomy" OR "PPPD"):ti,ab,kw  #3 #1 OR #2  #4 MeSH descriptor: [Somatostatin] explode all trees  #5 ("somatostatin" OR "somatostatin analogues" OR "octreotide" OR "lanreotide" OR "pasireotide" OR "vapreotide"):ti,ab,kw  #6 #4 OR #5  #7 MeSH descriptor: [Randomized Controlled Trial] explode all trees  #8 ("randomized controlled trial" OR "RCT" OR "randomized " OR "randomised " OR "random"):ti,ab,kw  #9 #7 OR #8  #10 #3 AND #6 AND #9  #11 (Review OR Systematic Reviews OR Meta-analysis OR Editorial OR Case Series OR Comment):ti  #12 #10 NOT #11 |
| EMBASE | No. Query  #1 'pancreatectomy'/exp OR 'pancreatectomy' OR 'pancreas':ab,ti OR 'pancreatic cancer':ab,ti OR 'pancreatic surgery':ab,ti OR 'pancreatectomy':ab,ti OR 'distal pancreatectomy':ab,ti OR 'whipple':ab,ti OR 'pancreaticoduodenectomy':ab,ti OR 'pancreatic resection':ab,ti OR 'total pancreatectomy':ab,ti OR 'partial pancreatectomy':ab,ti OR 'pylorus-preserving pancreaticoduodenectomy':ab,ti OR 'pppd':ab,ti  #2 'somatostatin'/exp OR 'somatostatin':ab,ti OR 'somatostatin analogues':ab,ti OR 'octreotide':ab,ti OR 'lanreotide':ab,ti OR 'pasireotide':ab,ti OR 'vapreotide':ab,ti  #3 'randomized controlled trial'/exp OR 'randomized controlled trial':ab,ti OR 'rct':ab,ti OR 'randomized':ab,ti OR 'randomised':ab,ti OR 'random':ab,ti  #4 'review'/exp OR 'meta analysis'/exp OR 'editorial'/exp OR 'case report'/exp OR 'pregnancy'/exp  #5 #1 AND #2 AND #3  #6 #5 NOT #4 |
| Web of Science | # Search Query  1 "((TS=(Pancreatectomy)) OR TI=(pancreas or pancreatic cancer or pancreatic surgery or Pancreatectomy or distal pancreatectomy or Whipple or Pancreaticoduodenectomy or pancreatic resection or Total pancreatectomy or Partial pancreatectomy or Pylorus-preserving pancreaticoduodenectomy or PPPD)) OR AB=(pancreas or pancreatic cancer or pancreatic surgery or Pancreatectomy or distal pancreatectomy or Whipple or Pancreaticoduodenectomy or pancreatic resection or Total pancreatectomy or Partial pancreatectomy or Pylorus-preserving pancreaticoduodenectomy or PPPD) "  2 "((TS=(Somatostatin)) OR TI=(somatostatin or somatostatin analogues or octreotide or lanreotide or pasireotide or vapreotide)) OR AB=(somatostatin or somatostatin analogues or octreotide or lanreotide or pasireotide or vapreotide) "  3 "(TS=(randomized controlled trial )) OR TI=(randomized controlled trial OR RCT OR randomized OR randomised OR random ) "  4 "(DT=(""Review"" OR ""Systematic Review"" OR ""Meta-Analysis"" OR ""Editorial Article"" OR ""Case Report"" OR ""Letter"")) "  5 "#3 AND #2 AND #1 "  6 "#5 NOT #4 " |
| OVID | 1 (" pancreas" or "pancreatic cancer" or "pancreatic surgery" or "Pancreatectomy" or "distal pancreatectomy" or "Whipple" or "Pancreaticoduodenectomy" or "pancreatic resection" or "Total pancreatectomy" or "Partial pancreatectomy" or "Pylorus-preserving pancreaticoduodenectomy" or "PPPD").ti,ab.  2 ("somatostatin" or "somatostatin analogues" or "octreotide" or "lanreotide" or "pasireotide" or "vapreotide").ti,ab.  3 ("randomized controlled trial" or "RCT" or "randomized " or "randomised " or "random").ti,ab.  4 ("Review" or "Systematic Review" or "Meta-Analysis" or "Editorial" or "Case Reports" or "Comment").pt.  5 1 and 2 and 3  6 (((" pancreas" or "pancreatic cancer" or "pancreatic surgery" or "Pancreatectomy" or "distal pancreatectomy" or "Whipple" or "Pancreaticoduodenectomy" or "pancreatic resection" or "Total pancreatectomy" or "Partial pancreatectomy" or "Pylorus-preserving pancreaticoduodenectomy" or "PPPD") and ("somatostatin" or "somatostatin analogues" or "octreotide" or "lanreotide" or "pasireotide" or "vapreotide") and ("randomized controlled trial" or "RCT" or "randomized " or "randomised " or "random")) not ("Review" or "Systematic Review" or "Meta-Analysis" or "Editorial" or "Case Reports" or "Comment")).af. |
| Scopus | ( ( TITLE-ABS-KEY ( "Drain" OR "drainage" ) ) AND ( TITLE-ABS-KEY ( "Pancreatectomy" OR "distal pancreatectomy" OR "Whipple" OR "Pancreaticoduodenectomy" OR "pancreatic resection" OR "Total pancreatectomy" OR "Partial pancreatectomy" OR "Pylorus-preserving pancreaticoduodenectomy" OR "PPPD" ) ) ) AND NOT ( DOCTYPE ( "rev" OR "srev" OR "meta" OR "ed" OR "cr" OR "com" ) ) |
