# Supplementary material for: The influence of somatostatin analogues on the incidence of pancreatic fistulas and postoperative morbidity in patients undergoing pancreatic resection: A Bayesian network meta-analysis
Source: PLoS One. 2025 Sep 19;20(9):e0331909. doi: 10.1371/journal.pone.0331909 (PMC12449010; doi:10.1371/journal.pone.0331909)
Supplement: S1 File — S1 Fig. Quality assessment of the included studies and risk of bias summary. S2 Fig. Funnel Char Of Publication Bias. A:POPF;B:CR-POPF;C:Mortality;D:Morbidity. S3 Fig. Forest plot for inconsistency testing.A:POPF;B:CR-POPF;C:ortality;D:Morbidity. S1 Table. PRISMA 2020 checklist. S2 Table.Index and keyword terms used in the databases. S3 Table.Lists of clinical trial registries and specialized journals. S4 Table.Eligibility criteria. S5 Table.Specific meaning of certainty in effect estimates. S6 Table.List of excluded studies. S7 Table.GRADE Quality Assessment Table for Network Analysis Results. S8 Table The dataset utilized for the purposes of this investigation. S9 Table Sensitivity Analysis. (ZIP) [file pone.0331909.s001.zip › S3_Table.docx]

# **S3 Table.** Lists of clinical trial registries and specialized journals.

| Sources | Website |
| --- | --- |
| ***Clinical trial registries:*** |  |
| Australian New Zealand Clinical Trials Registry | http://www.anzctr.org.au/ |
| CenterWatch | http://www.centerwatch.com/ |
| ClinicalTrials.gov | https://clinicaltrials.gov/ |
| Cochrane Central Register of Controlled Trials | https://www.cochranelibrary.com/central/about-central |
| EU Clinical Trials Register | https://www.clinicaltrialsregister.eu/ctr-search/search |
|  |  |
| Singapore Clinical Trials Register | https://www.hsa.gov.sg/clinical-trials/clinical-trials-register |
| UK Clinical Research Network: Portfolio Database | https://www.nihr.ac.uk/research-and-impact/nihr-clinical-research-network-portfolio/ |
| World Health Organization International Clinical Trials Registry Platform  European Medicines Agency  International Standard Randomised Controlled Trial Number Registry | https://www.who.int/ictrp  https://www.ema.europa.eu/ema/  https://www.isrctn.com/ |
| ***Specialized journals:*** |  |
| Annals of Surgery | https://journals.lww.com/annalsofsurgery/pages/default.aspx |
| BMC Medical Informatics and Decision Making | https://bmcmedinformdecismak.biomedcentral.com/ |
| British Journal of Surgery | https://bjssjournals.onlinelibrary.wiley.com/journal/13652168 |
| International Journal of Surgery | https://www.journals.elsevier.com/international-journal-of-surgery |
| JAMA Surgery | https://jamanetwork.com/journals/jamasurgery |
| Journal of the American College of Surgeons | https://www.journalacs.org/ |
| Journal of Vascular Surgery | https://www.jvascsurg.org/ |
| Medical Decision Making | https://journals.sagepub.com/home/mdm |
| Plastic and Reconstructive Surgery | https://journals.lww.com/plasreconsurg/pages/default.aspx |
| Surgery | https://www.journals.elsevier.com/surgery |
| The American Journal of Surgery | https://www.journals.elsevier.com/the-american-journal-of-surgery |
| World Journal of Surgery | https://www.springer.com/journal/268 |
|  |  |
| ***Grey literature resources:*** |  |
| CogPrints | http://cogprints.org/ |
| Google Scholar | http://scholar.google.com.sg/ |
| GreySource | http://www.greynet.org/greysourceindex.html |
| NYAM Grey Literature Report | http://www.greylit.org/ |
| OpenGrey: System for Information on Grey Literature in Europe | http://www.opengrey.eu/ |
| OpenMD.com | https://openmd.com/directory/allergy-immunology |
| Science.gov | http://www.science.gov/scigov/ |
| World Bank Publications | https://www.worldbank.org/en/research |
