# Supplementary material for: The influence of somatostatin analogues on the incidence of pancreatic fistulas and postoperative morbidity in patients undergoing pancreatic resection: A Bayesian network meta-analysis
Source: PLoS One. 2025 Sep 19;20(9):e0331909. doi: 10.1371/journal.pone.0331909 (PMC12449010; doi:10.1371/journal.pone.0331909)
Supplement: S1 File — S1 Fig. Quality assessment of the included studies and risk of bias summary. S2 Fig. Funnel Char Of Publication Bias. A:POPF;B:CR-POPF;C:Mortality;D:Morbidity. S3 Fig. Forest plot for inconsistency testing.A:POPF;B:CR-POPF;C:ortality;D:Morbidity. S1 Table. PRISMA 2020 checklist. S2 Table.Index and keyword terms used in the databases. S3 Table.Lists of clinical trial registries and specialized journals. S4 Table.Eligibility criteria. S5 Table.Specific meaning of certainty in effect estimates. S6 Table.List of excluded studies. S7 Table.GRADE Quality Assessment Table for Network Analysis Results. S8 Table The dataset utilized for the purposes of this investigation. S9 Table Sensitivity Analysis. (ZIP) [file pone.0331909.s001.zip › S6_Table.docx]

# **S6 Table.** List of excluded studies.

| **Year** | **Title** | **DOI/**PMID | **Reason for exclusion** |
| --- | --- | --- | --- |
| 1992 | A randomized, prospective trial of postoperative somatostatin analogue in patients with neuroendocrine tumors of the pancreas | PMID: 1455306 | This study is a abstract. |
| 1993 | Perioperative use of somatostatin in pancreatic surgery | PMID: 7527608 | This study is a abstract. |
| 1995 | Somatostatin prophylaxis following cephalic duodenopancreatectomy | PMID: 7742052 | This study is a abstract. |
| 1998 | Prospective and randomized study on the usefulness of octreotide in the prevention of complications after cephalic duodeno-pancreatectomy | PMID: 9824933 | This study is a abstract. |
| 2009 | 817 Does Octreotide Has Any Beneficial Effect in Patients with High Risk to Develop Pancreatic Fistula After Pancreaticoduodenectomy: A Prospective Randomized Trial | [10.1016/S0016-5085(09)64070-1](http://dx.doi.org/10.1016/S0016-5085(09)64070-1" \o "http://dx.doi.org/10.1016/S0016-5085(09)64070-1) | This study is a abstract. |
| 2012 | Does preoperative somatostatin administration decrease the rate of postoperative pancreatic fistula following pancreaticoduodenectomy? A randomized placebo-controlled trial | 10.1111/jgh.12006 | This study is a abstract. |
| 2014 | Pasireotide for prevention of pancreatic leak: is there light at the end of the tunnel? | 10.1002/central/CN-01091218/full | This study is a abstract. |
| 2018 | Impact of prophylactic octreotide to pancreatic exocrine secretion and pancreatic fistula after pancreatoduodenectomy: RCT | 10.1016/j.hpb.2018.06.2271 | This study is a abstract. |
| 2021 | Somatostatin Prevents Clinically Relevant Pancreatic Fistula in Intermediate Risk Patients after Pancreaticoduodenectomy (SPEED): a Multi-center, Randomized, Controlled Study | 10.1016/j.hpb.2020.11.158 | This study is a abstract. |
| 2023 | Somatostatin vs Octreotide for Postoperative Pancreatic Fistula Prevention - A Prospective Randomized Controlled Study - A FRENCH 007 – ACHBT Study | 10.1016/j.hpb.2023.07.163 | This study is a abstract. |
| 2001 | Does prophylactic octreotide decrease the rates of pancreatic fistula and other complications after pancreaticoduodenectomy? | 10.1097/00000658-200108000-00017 |  |
| 1996 | Inhibition of secretion in pancreas surgery reduces postoperative complication rate: a meta analysis of 4 multicenter studies | PMID: 9101848 | This study is a meta. |
| 2007 | Use of octreotide for the prevention of pancreatic fistula after elective pancreatic surgery: A systematic review and meta-analysis | PMID: 18053374 | This study is a meta. |
| 2008 | Efficacy of somatostatin and its analogues in prevention of postoperative complications after pancreaticoduodenectomy: A meta-analysis of randomized controlled trials | 10.1097/mpa.0b013e3181343f5d | This study is a meta. |
| 2010 | Meta-analysis of randomized controlled trials on the effectiveness of somatostatin analogues for pancreatic surgery: A Cochrane review | 10.1111/j.1477-2574.2010.00157.x | This study is a meta. |
| 2012 | Systematic review and meta-analysis of somatostatin analogues for the treatment of pancreatic fistula | 10.1002/bjs.8709 | This study is a meta. |
| 2013 | Somatostatin analogues for pancreatic surgery | 10.1002/14651858.CD008370.pub3 | This study is a meta. |
| 2015 | Systematic review and meta-analysis of somatostatin analogues in the prevention of postoperative complication after pancreaticoduodenectomy | 10.1159/000381032 | This study is a meta. |
| 2017 | The effect of somatostatin analogues on postoperative outcomes following pancreatic surgery: A meta-analysis | 10.1371/journal.pone.0188928 | This study is a meta. |
| 2018 | The Role of Prophylactic Octreotide Following Pancreaticoduodenectomy to Prevent Postoperative Pancreatic Fistula: A Meta-Analysis of the Randomized Controlled Trials | 10.1055/s-0038-1675359 | This study is a meta. |
| 2019 | The Use of Prophylactic Somatostatin Therapy Following Pancreaticoduodenectomy: A Meta-analysis of Randomised Controlled Trials | 10.1007/s00268-019-04956-6 | This study is a meta. |
| 2019 | An updated systematic review and meta-analysis of the use of octreotide for the prevention of postoperative complications after pancreatic resection | 10.1097/MD.0000000000017196 | This study is a meta. |
| 2020 | Meta-Analysis on the Effect of Pasireotide for Prevention of Postoperative Pancreatic Fistula | 10.1177/0003134820947371 | This study is a meta. |
| 2020 | Do somatostatin-analogues have the same impact on postoperative morbidity and pancreatic fistula in patients after pancreaticoduodenectomy and distal pancreatectomy? – A systematic review with meta-analysis of randomized-controlled trials | 10.1016/j.pan.2020.10.043 | This study is a meta. |
| 2006 | Somatostatin analogues in the prevention of pancreas-related complications after pancreatic resection | 10.1007/s00534-005-1033-9 | This study cannot obtain data. |
| 2012 | Influences of Somatostatin Analogues on the Morbidity of Pancreatic Fistula After Pancreaticojejunostomy: a Randomized, Double-blind, Placebo-controlled Trial | 10.1002/central/CN-01821475/full | This study cannot obtain data. |
| 1992 | Somatostatin in duodenocephalopancreatectomy for neoplastic pathology | PMID: 1351276 | This study cannot obtain the full text. |
| 1996 | The role of octreotide in the prevention of pancreatic fistula after pancreaticoduodenectomy for periampullary malignancy: A prospective randomized trial | https://journals.lww.com/annalsofsurgery/abstract/1997/11000/prospective,_randomized_trial_of_octreotide_to.8.aspx | This study cannot obtain the full text. |
| 1998 | A prospective-randomized trial using Octreotide for prevention of complications following pancreaticoduodenectomy | 10.1002/central/CN-01746726/full | This study cannot obtain the full text. |
| 2000 | Prevention of intra-abdominal complications after pancreatic resection by octreotide. A prospective, multicenter, randomized trial | 10.1016/S0016-5085(00)83965-7 | This study cannot obtain the full text. |
| 2005 | Prospectively randomized trial using perioperative low dose octreotide to prevent organ related and general complications following pancreatic surgery and pancreatico-jejunostomy | 10.1080/00015458.2005.11679741 | This study cannot obtain the full text. |
| 2008 | Randomized controlled trial comparing somatostatin with octreotide in the prevention of complications after pancreatectomy | 10.1002/central/CN-00666510/full | This study cannot obtain the full text. |
| 2012 | A prospective randomized controlled trial on use of octreotide in patients with soft pancreas undergoing pancreaticoduodenectomy: interim analysis | 10.1111/j.1477-2574.2012.00511.x | This study cannot obtain the full text. |
| 2013 | A randomized, double-blind, placebo-Controlled study of Lanreotide Antiproliferative Response in patients with gastroenteropancreatic NeuroEndocrine Tumors (CLARINET) | 10.1016/S0959-8049(13)70069-8 | This study cannot obtain the full text. |
| 2013 | Randomized, placebo-controlled study of the efficacy of preoperative somatostatin administration in the prevention of postoperative complications following pancreaticoduodenectomy | 10.5754/hge12669 | This study cannot obtain the full text. |
| 1999 | Economic evaluation of the use of octreotide for prevention of complications following pancreatic resection | 10.1016/s1091-255x(99)80064-x | This research is not an RCT study. |
| 2014 | A Cost Analysis of Somatostatin use in the Prevention of Pancreatic Fistula after Pancreatectomy | 10.1007/s00268-014-2512-4 | This research is not an RCT study. |
| 2014 | Preliminary results using preoperative long-acting somatostatin analogue in pancreaticoduodenectomy with pancreaticogastrostomy | https://www.embase.com/search/results?subaction=viewrecord&id=L71383131&from=export | This research is not an RCT study. |
| 2020 | Pasireotide administration after pancreaticoduodenectomy may decrease clinically relevant postoperative pancreatic fistula in high-risk patients with small pancreatic ducts, soft pancreatic parenchyma and cystic or neuroendocrine neoplasia | https://dx.doi.org/10.1016/j.pan.2020.03.010 | This research is not an RCT study. |
| 2021 | Lanreotide for the prevention of postoperative pancreatic fistula: phase II clinical trial results | 10.1016/j.hpb.2021.06.062 | This research is not an RCT study. |
| 2022 | Lanreotide for the Prevention of Postoperative Pancreatic Fistula: phase Ii Clinical Trial Results | 10.1016/j.hpb.2022.05.820 | This research is not an RCT study. |
| 2022 | A phase II trial of lanreotide for the prevention of postoperative pancreatic fistula | 10.1016/j.hpb.2022.07.011 | This research is not an RCT study. |
| 1991 | Effect of somatostatin on basal and stimulated exocrine pancreatic secretion after partial duodenopancreatectomy. A clinical experimental study | PMID: 1677616 | Not relevant to our study |
| 1997 | The effect of octreotide on gastric emptying at a dosage used to prevent complications after pancreatic surgery: a randomised, placebo controlled study in volunteers | 10.1136/gut.41.6.758 | Not relevant to our study |
| 2017 | The Cost of Postoperative Pancreatic Fistula Versus the Cost of Pasireotide: Results from a Prospective Randomized Trial | 10.1097/sla.0000000000001892 | Not relevant to our study |
| 2020 | The effect of using long-acting octreotide as adjuvant therapy for patients with grade 2 pancreatic neuroendocrine tumors after radical resection | 10.1097/JP9.0000000000000058 | Not relevant to our study |
| 2020 | Study Protocol of the PreFiPS Study: Prevention of Postoperative Pancreatic Fistula by Somatostatin Compared With Octreotide, a Prospective Randomized Controlled Trial | 10.3389/fmed.2020.00488 | Not relevant to our study |
| 2021 | Study Protocol of the PreFiPS Study: Prevention of Postoperative Pancreatic Fistula by Somatostatin Compared With Octreotide, a Prospective Randomized Controlled Trial | 10.3389/fmed.2020.00488 | Not relevant to our study |
| 2021 | Effect of intraoperative secretin on operative outcomes in pancreatic resection: A randomized controlled trial | 10.1016/j.pan.2021.02.002 | Not relevant to our study |
| 2016 | How Much Should We Pay to Minimize Pancreatic Leak? The Cost-effectiveness of Pasireotide in Pancreatic Resection: RETRACTED | 10.1097/SLA.0000000000001411 | The abstract was retracted. |
| 1999 | The role of octreotide in the prevention of complications following pancreatic resection | 10.1159/000051476 | Review article |
| 2001 | Somatostatin and octreotide in the prevention of postoperative pancreatic complications and the treatment of enterocutaneous pancreatic fistulas: A systematic review of randomized controlled trials | 10.1046/j.1365-2168.2001.01659.x | Review article |
| 2012 | The effect of somatostatin and its analogs in the prevention of pancreatic fistula after elective pancreatic surgery | 10.1007/s10353-011-0612-z | Review article |
