# Supplementary material for: The influence of somatostatin analogues on the incidence of pancreatic fistulas and postoperative morbidity in patients undergoing pancreatic resection: A Bayesian network meta-analysis
Source: PLoS One. 2025 Sep 19;20(9):e0331909. doi: 10.1371/journal.pone.0331909 (PMC12449010; doi:10.1371/journal.pone.0331909)
Supplement: S1 File — S1 Fig. Quality assessment of the included studies and risk of bias summary. S2 Fig. Funnel Char Of Publication Bias. A:POPF;B:CR-POPF;C:Mortality;D:Morbidity. S3 Fig. Forest plot for inconsistency testing.A:POPF;B:CR-POPF;C:ortality;D:Morbidity. S1 Table. PRISMA 2020 checklist. S2 Table.Index and keyword terms used in the databases. S3 Table.Lists of clinical trial registries and specialized journals. S4 Table.Eligibility criteria. S5 Table.Specific meaning of certainty in effect estimates. S6 Table.List of excluded studies. S7 Table.GRADE Quality Assessment Table for Network Analysis Results. S8 Table The dataset utilized for the purposes of this investigation. S9 Table Sensitivity Analysis. (ZIP) [file pone.0331909.s001.zip › S7_Table.docx]

# **S7.1 Table.** GRADE Quality Assessment Table for Network Analysis Results.

| **complication** | **Drug comparison** | **Study  limitations** | **Imprecision** | **Inconsistency** | **Indirectness** | **Publication bias** | **GRADE** |
| --- | --- | --- | --- | --- | --- | --- | --- |
| **POPF** | placebo vs octreotide | Downgrade | No downgrade | No downgrade | No downgrade | No downgrade | MODERATE |
|  | placebo vs somatostatin | Downgrade | Downgrade | No downgrade | No downgrade | No downgrade | LOW |
|  | placebo vs vapreotide | Downgrade | Downgrade | No downgrade | No downgrade | Downgrade | VERY LOW |
|  | placebo vs pasireotid | Downgrade | No downgrade | No downgrade | No downgrade | Downgrade | LOW |
|  | octreotide vs somatostatin | Downgrade | Downgrade | No downgrade | No downgrade | No downgrade | LOW |
|  | octreotide vs vapreotide | Downgrade | Downgrade | No downgrade | No downgrade | Downgrade | VERY LOW |
|  | octreotide vs pasireotid | Downgrade | Downgrade | No downgrade | No downgrade | Downgrade | VERY LOW |
|  | somatostatin vs vapreotide | Downgrade | Downgrade | No downgrade | No downgrade | Downgrade | VERY LOW |
|  | somatostatin vs pasireotid | Downgrade | Downgrade | No downgrade | No downgrade | Downgrade | VERY LOW |
|  | vapreotide vs pasireotid | Downgrade | No downgrade | No downgrade | No downgrade | Downgrade | LOW |
| **CR-POPF** | placebo vs octreotide | Downgrade | No downgrade | No downgrade | No downgrade | No downgrade | MODERATE |
|  | placebo vs somatostatin | Downgrade | No downgrade | No downgrade | No downgrade | No downgrade | MODERATE |
|  | placebo vs pasireotid | Downgrade | No downgrade | No downgrade | No downgrade | Downgrade | LOW |
|  | octreotide vs somatostatin | Downgrade | Downgrade | No downgrade | No downgrade | No downgrade | LOW |
|  | octreotide vs pasireotid | Downgrade | Downgrade | No downgrade | No downgrade | Downgrade | VERY LOW |
|  | somatostatin vs pasireotid | Downgrade | Downgrade | No downgrade | No downgrade | Downgrade | VERY LOW |
| **Mortality** | placebo vs octreotide | Downgrade | Downgrade | No downgrade | No downgrade | No downgrade | LOW |
|  | placebo vs somatostatin | Downgrade | Downgrade | No downgrade | No downgrade | No downgrade | LOW |
|  | placebo vs vapreotide | Downgrade | No downgrade | No downgrade | No downgrade | Downgrade | LOW |
|  | placebo vs pasireotid | Downgrade | Downgrade | No downgrade | No downgrade | Downgrade | VERY LOW |
|  | octreotide vs somatostatin | Downgrade | Downgrade | No downgrade | No downgrade | No downgrade | LOW |
|  | octreotide vs vapreotide | Downgrade | No downgrade | No downgrade | No downgrade | Downgrade | LOW |
|  | octreotide vs pasireotid | Downgrade | Downgrade | No downgrade | No downgrade | Downgrade | VERY LOW |
|  | somatostatin vs vapreotide | Downgrade | No downgrade | No downgrade | No downgrade | Downgrade | LOW |
|  | somatostatin vs pasireotid | Downgrade | Downgrade | No downgrade | No downgrade | Downgrade | VERY LOW |
|  | vapreotide vs pasireotid | Downgrade | No downgrade | No downgrade | No downgrade | Downgrade | LOW |
| **Morbidity** | placebo vs octreotide | Downgrade | No downgrade | No downgrade | No downgrade | No downgrade | MODERATE |
|  | placebo vs somatostatin | Downgrade | No downgrade | No downgrade | No downgrade | No downgrade | MODERATE |
|  | placebo vs vapreotide | Downgrade | Downgrade | No downgrade | No downgrade | Downgrade | VERY LOW |
|  | placebo vs pasireotid | Downgrade | Downgrade | No downgrade | No downgrade | Downgrade | VERY LOW |
|  | octreotide vs somatostatin | Downgrade | Downgrade | No downgrade | No downgrade | No downgrade | LOW |
|  | octreotide vs vapreotide | Downgrade | No downgrade | No downgrade | No downgrade | Downgrade | LOW |
|  | octreotide vs pasireotid | Downgrade | No downgrade | No downgrade | No downgrade | Downgrade | LOW |
|  | somatostatin vs vapreotide | Downgrade | No downgrade | No downgrade | No downgrade | Downgrade | LOW |
|  | somatostatin vs pasireotid | Downgrade | No downgrade | No downgrade | No downgrade | Downgrade | LOW |
|  | vapreotide vs pasireotid | Downgrade | Downgrade | No downgrade | No downgrade | Downgrade | MODERATE |

# **S7.2 Table.** GRADE Quality Assessment Table for Network Analysis Results In PD-subgroup

| **complication** | **Drug comparison** | **Study  limitations** | **Imprecision** | **Inconsistency** | **Indirectness** | **Publication bias** | **GRADE** |
| --- | --- | --- | --- | --- | --- | --- | --- |
| **POPF** | placebo vs octreotide | Downgrade | Downgrade | Downgrade | No downgrade | No downgrade | VERY LOW |
|  | placebo vs somatostatin | Downgrade | Downgrade | Downgrade | No downgrade | No downgrade | VERY LOW |
|  | octreotide vs somatostatin | Downgrade | Downgrade | Downgrade | No downgrade | No downgrade | VERY LOW |
| **CR-POPF** | placebo vs octreotide | Downgrade | Downgrade | No downgrade | No downgrade | No downgrade | LOW |
|  | placebo vs somatostatin | Downgrade | No downgrade | No downgrade | No downgrade | No downgrade | MODERATE |
|  | octreotide vs somatostatin | Downgrade | Downgrade | No downgrade | No downgrade | No downgrade | LOW |
| **Mortality** | placebo vs octreotide | Downgrade | Downgrade | Downgrade | No downgrade | No downgrade | VERY LOW |
|  | placebo vs somatostatin | Downgrade | Downgrade | Downgrade | No downgrade | No downgrade | VERY LOW |
|  | octreotide vs somatostatin | Downgrade | Downgrade | Downgrade | No downgrade | No downgrade | VERY LOW |
| **Morbidity** | placebo vs octreotide | Downgrade | Downgrade | Downgrade | No downgrade | No downgrade | VERY LOW |
|  | placebo vs somatostatin | Downgrade | Downgrade | Downgrade | No downgrade | No downgrade | VERY LOW |
|  | octreotide vs somatostatin | Downgrade | Downgrade | No downgrade | No downgrade | No downgrade | LOW |

Each network estimate was rated according to the following criteria:①.Research Limitations: Given that all studies were not of low risk, all outcome measures were adjusted accordingly.②.Inaccuracy: We deemed a clinically significant threshold for the Relative Risk (RR) to be 0.80 or 1.25. If the point estimate of RR was 1 or higher, but the lower bound of its Confidence Interval (CrI) fell below 0.80; or if the point estimate of OR was less than 1, but the upper bound of its confidence interval exceeded 1.25, we downgraded the estimate.③.Inconsistency: In this field, we assessed two concepts, heterogeneity and discordance (inconsistency). For heterogeneity, we observed common I2 values, all less than 50%, hence we did not downgrade any network estimates due to heterogeneity. For discordance, we calculated p-values using the node splitting method, with results indicating that all p-values were greater than 0.05, thus we did not downgrade any estimates due to inconsistency.④.Indirectness: All studies involved patients who had undergone pancreatectomy, and all outcome measures were obtained directly and objectively, hence we did not downgrade any estimates due to indirectness.⑤Publication Bias: Our research team essentially covered all major databases, with sufficient numbers of studies on somatostatin and octreotide, and no significant deviation observed in the funnel plot. However, only one study was available for vapreotide and pasireotid, and the review team decided to downgrade the publication bias for all outcomes compared to potential vapreotide and pasireotid by one level as a default.
