# Supplementary material for: The influence of somatostatin analogues on the incidence of pancreatic fistulas and postoperative morbidity in patients undergoing pancreatic resection: A Bayesian network meta-analysis
Source: PLoS One. 2025 Sep 19;20(9):e0331909. doi: 10.1371/journal.pone.0331909 (PMC12449010; doi:10.1371/journal.pone.0331909)
Supplement: S1 File — S1 Fig. Quality assessment of the included studies and risk of bias summary. S2 Fig. Funnel Char Of Publication Bias. A:POPF;B:CR-POPF;C:Mortality;D:Morbidity. S3 Fig. Forest plot for inconsistency testing.A:POPF;B:CR-POPF;C:ortality;D:Morbidity. S1 Table. PRISMA 2020 checklist. S2 Table.Index and keyword terms used in the databases. S3 Table.Lists of clinical trial registries and specialized journals. S4 Table.Eligibility criteria. S5 Table.Specific meaning of certainty in effect estimates. S6 Table.List of excluded studies. S7 Table.GRADE Quality Assessment Table for Network Analysis Results. S8 Table The dataset utilized for the purposes of this investigation. S9 Table Sensitivity Analysis. (ZIP) [file pone.0331909.s001.zip › S8_Table.docx]

|  |  |  |  |  | popf | | | | crpopf | | | | mortality | | | | morbidity | | | |
| --- | --- | --- | --- | --- | --- | --- | --- | --- | --- | --- | --- | --- | --- | --- | --- | --- | --- | --- | --- | --- |
|  |  |  |  |  | a | | b | | a | | b | | a | | b | | a | | b | |
| ID | **Study** | **year** | **a** | **b** | e.e | e.t | c.e | c.t | e.e | e.t | c.e | c.t | e.e | e.t | c.e | c.t | e.e | e.t | c.e | c.t |
| 1 | Büchler | 1992 | octreotide | placebo | 22 | 125 | 46 | 121 |  |  |  |  | 4 | 125 | 7 | 121 | 40 | 125 | 67 | 121 |
| 2 | Pederzoli | 1994 | octreotide | placebo | 11 | 122 | 24 | 130 |  |  |  |  | 2 | 122 | 5 | 130 | 19 | 122 | 38 | 130 |
| 3 | Friess | 1995 | octreotide | placebo | 12 | 122 | 28 | 125 |  |  |  |  | 2 | 122 | 1 | 125 | 20 | 122 | 37 | 125 |
| 4 | Montorsi | 1995 | octreotide | placebo | 10 | 111 | 21 | 107 |  |  |  |  | 9 | 111 | 6 | 107 | 36 | 111 | 65 | 107 |
| 5 | Lowy | 1997 | octreotide | placebo | 16 | 57 | 11 | 53 | 9 | 57 | 8 | 53 | 1 | 57 | 0 | 53 | 17 | 57 | 13 | 53 |
| 6 | Benedetti | 1998 | octreotide | placebo |  |  |  |  |  |  |  |  | 1 | 10 | 2 | 7 |  |  |  |  |
| 7 | Yeo | 2000 | octreotide | placebo | 11 | 104 | 10 | 107 |  |  |  |  | 1 | 104 | 0 | 107 | 42 | 104 | 36 | 107 |
| 8 | Gouillat | 2001 | somatosta | placebo | 4 | 38 | 10 | 37 | 2 | 38 | 8 | 37 | 2 | 38 | 1 | 37 | 5 | 38 | 12 | 37 |
| 9 | Shan | 2003 | somatosta | placebo | 2 | 27 | 2 | 27 |  |  |  |  | 1 | 27 | 1 | 27 | 7 | 27 | 14 | 27 |
| 10 | Sarr | 2003 | vapreotide | placebo | 32 | 135 | 32 | 140 |  |  |  |  | 0 | 135 | 2 | 140 | 41 | 135 | 37 | 140 |
| 11 | Suc | 2004 | octreotide | NA | 21 | 122 | 20 | 108 |  |  |  |  | 15 | 122 | 7 | 108 | 27 | 122 | 35 | 108 |
| 12 | Hesse | 2005 | octreotide | NA |  | 55 | 4 | 50 |  |  |  |  | 1 | 55 | 0 | 50 | 6 | 55 | 6 | 50 |
| 13 | Shan | 2005 | somatostati | NA | 2 | 11 | 0 | 12 |  |  |  |  | 0 | 11 | 1 | 12 |  |  |  |  |
| 14 | Kollmar | 2008 | octreotide | placebo | 0 | 35 | 1 | 32 |  |  |  |  | 2 | 35 | 1 | 32 |  |  |  |  |
| 15 | Katsourakis | 2010 | somatostatin | NA | 1 | 35 | 5 | 32 |  |  |  |  |  |  |  |  | 7 | 35 | 19 | 32 |
| 16 | **Fernández-Cruz** | **2013** | **octreotide** | placebo |  |  |  |  | 2 | 32 | 3 | 30 | 0 | 32 | 0 | 30 | 10 | 32 | 11 | 30 |
| 17 | Katsourakis | 2013 | somatostatin-14 | NA | 1 | 35 | 5 | 32 |  |  |  |  | 1 | 35 | 2 | 32 | 6 | 35 | 18 | 32 |
| 18 | Allen | 2014 | pasireotid | placebo | 12 | 152 | 25 | 148 | 12 | 152 | 25 | 146 | 1 | 152 | 1 | 148 | 140 | 152 | 133 | 148 |
| 19 | Kurumbo | 2015 | octreotide | NA | 33 | 55 | 34 | 54 | 6 | 55 | 10 | 54 | 1 | 55 | 1 | 54 | 10 | 55 | 16 | 54 |
| 20 | Kong | 2016 | octreotide | placebo | 18 | 147 | 25 | 159 | 5 | 147 | 9 | 159 | 5 | 147 | 7 | 159 | 44 | 147 | 51 | 159 |
| 21 | El Nakeeb | **2018** | **octreotide** | placebo | 10 | 52 | 11 | 52 | 4 | 52 | 4 | 52 | 3 | 52 | 3 | 52 | 20 | 52 | 19 | 52 |
| 22 | You | 2019 | octreotide | NA | 7 | 29 | 15 | 30 | 1 | 29 | 5 | 30 | 7 | 29 | 5 | 30 |  |  |  |  |
| 23 | Cao | 2021 | somatostatin | NA | 51 | 99 | 44 | 100 |  |  |  |  | 0 | 99 | 1 | 100 | 62 | 99 | 57 | 100 |
| 24 | Gaujoux | 2024 | Somatostatin | Octreotide | 168 | 327 | 158 | 324 | 78 | 327 | 75 | 324 | 9 | 327 | 8 | 324 | 183 | 327 | 179 | 324 |
